# Preferences in Information Processing, Marginalized Identity, and Non-Monogamy: Understanding Factors in Suicide-Related Behavior among Members of the Alternative Sexuality Community

**DOI:** 10.3390/ijerph17093233

**Published:** 2020-05-06

**Authors:** Robert J. Cramer, Jennifer Langhinrichsen-Rohling, Andrea R. Kaniuka, Corrine N. Wilsey, Annelise Mennicke, Susan Wright, Erika Montanaro, Jessamyn Bowling, Kristin E. Heron

**Affiliations:** 1 Department of Public Health Sciences, UNC Charlotte, Charlotte, NC 28233 USA; akaniuka@uncc.edu (A.R.K.); cwilsey@uncc.edu (C.N.W.); jbowlin9@uncc.edu (J.B.); 2 Department of Psychological Sciences, UNC Charlotte, Charlotte, NC 28233, USA; jlanghin@uncc.edu (J.L.-R.); emontana@uncc.edu (E.M.); 3 School of Social Work, UNC Charlotte, Charlotte, NC 28233, USA; amennick@uncc.edu; 4 National Coalition for Sexual Freedom, Baltimore, MD 21202, USA; susan@ncsfreedom.org; 5 Department of Psychology, Old Dominion University, Norfolk, VA 23529, USA; kheron@odu.edu

**Keywords:** suicide, non-monogamy, Need for Affect, Need for Cognition, depression, information processing, national coalition for sexual freedom

## Abstract

Suicide-related behavior (SRB) is a mental health disparity experienced by the alternative sexuality community. We assessed mental health, relationship orientation, marginalized identities (i.e., sexual orientation minority, gender minority, racial minority, ethnic minority, and lower education), and preferences in information processing (PIP) as factors differentiating lifetime SRB groups. An online cross-sectional survey study was conducted in 2018. Members of the National Coalition for Sexual Freedom (NCSF; *n* = 334) took part. Bivariate analyses identified the following SRB risk factors: female and transgender/gender non-binary identity, sexual orientation minority identity, lower education, suicide attempt/death exposure, Need for Affect (NFA) Avoidance, depression, and anxiety. Monogamous relationship orientation was a protective factor. Multi-nomial regression revealed the following: (1) monogamous relationship orientation was a protective factor for suicidal ideation and attempt; (2) lower education was a risk factor for suicide attempt; (3) anxiety was a risk factor for suicide attempt; and (4) depression was a risk factor for suicidal ideation. A two-way interaction showed that elevated NFA Approach buffered the negative impacts of depression. Relationship orientation, several marginalized identities (i.e., based on gender, sexual orientation, and educational level), and PIP all contributed uniquely to SRB. Further study is necessary to understand the role of relationship orientation with suicide. Health education and suicide prevention efforts with NCSF should be tailored to account for marginalized identity, mental health, and NFA factors.

## 1. Introduction

### 1.1. Background

The National Coalition for Sexual Freedom (NCSF) was formed in 1997 in order to create “a political, legal, and social environment in the U.S. that advances equal rights for consenting adults who engage in alternative sexual and relationship expressions” [1]. Specifically, the aim of the organization is to advance the rights of, and advocate for consenting adults in the bondage, discipline, domination/submission, and sadomasochism (BDSM) lifestyle, and non-monogamy communities (heretofore referred to as *alternative sexuality community*). As part of their advocacy efforts, NCSF has supported research to understand and promote health among members of the alternative sexuality community. The ongoing research effort is important as this community is substantially understudied, and yet faces considerable societal misunderstanding, stigma, and discrimination [2,3,4]. Such stigma and discrimination are particularly concerning when viewed through a public health lens, as stigma has been identified as a core factor in the creation and maintenance of health disparities for a range of minority groups [5,6,7]. A particular focus on the mental health of the alternative sexuality community is consistent with models of health equity [8], because this subgroup comprises a diverse body with respect to other marginalized groups. For example, NCSF’s membership is composed of people with a wide range of sexual orientations (e.g., pansexual, heteroflexible), gender identities (e.g., gender queer, agender), and romantic relationship orientations (e.g., polyamory, open relationships) [9,10,11]. Although a full review of sexual orientation, gender identity, and romantic relationship orientation definitions is beyond the scope of the present paper, we recommend referral to the publicly available sexual and gender diversity glossaries for those unfamiliar with this area [10,11].

### 1.2. Suicide-Related Behavior among the Alternative Sexuality Community

One deleterious outcome prevalent among members of the alternative sexuality community garnering recent empirical attention is suicide-related behavior (SRB) [12,13]. SRB comprises both suicidal ideation and attempts [14,15]. Use of SRB phrasing is consistent with contemporary literature differentiating persons with subtypes of lifetime histories (e.g., ideation only vs. suicide attempt) and developing suicide interventions [16,17,18]. Past studies have examined alternative sexuality and SRB through the lens of one of the leading theories, namely the Interpersonal–Psychological Theory of Suicide [19]. Importantly, in a study of BDSM practicing adults, stigma-associated emotions of shame and guilt were found to be associated with suicidal ideation through feelings of perceived burdensomeness and thwarted belonging [13]. In that same sample, alternative sexuality identity and behavior were associated with lifetime suicide attempt as mediated by increased pain tolerance [12]. These findings lend credence to the importance of both stigma and gender (as a multiple marginalized factor) in understanding SRB among members of this community. Notably, the Interpersonal–Psychological Theory was not applied to other members of the NCSF community such as those in the polyamory community. A subsequent examination of suicide-related patterns and risk/protective factors among NCSF members found that: (1) NCSF members were at an elevated clinical risk for suicide compared to college student and general adult comparison groups [20]; and (2) among sexual orientation minority-identifying members of NCSF, symptoms of depression, post-traumatic stress, and impulsivity were prominent risk factors for total SRB [21]. These findings raise concern about suicide risk among NCSF members and highlight sexual orientation as another potential multiple marginalized identity factor worthy of further investigation.

Although this set of studies identified several risk factors for the alternative sexuality community and observed elevated clinical risk specifically for NCSF members, gaps remain. First, the literature has only begun to identify factors pertinent to suicidal ideation versus attempt, despite leading theories of suicide emphasizing such approaches [19,22]. A growing body of evidence indicates that there may be different risk and protective factors and processes that distinguish among non-suicidal individuals, those expressing suicidal ideation without attempt (ideation only), and those exhibiting a history of suicide attempt [23,24,25]. Importantly, some traditional markers for suicide (e.g., depression, hopelessness) differentiated individuals with suicidal ideation from non-suicidal individuals, but failed to distinguish between those with suicidal ideation versus suicide attempts [24,25]. The approach of comparing among the three groups (people who are non-suicidal, have suicidal ideation, and who have attempted suicide) is consistent with the ideation-to-action framework, which emphasizes the identification of risk factors for the transition from suicide ideation to suicide attempt [26]. The present study applies the distinction of non-suicidal, ideation only, and attempt subgroupings to understanding SRB among alternative sexuality community members. A second gap in the study of suicide among this population is the absence of identification of protective factors. The importance of population-specific protective factors cannot be understated. Such factors can serve as targets for prevention and intervention programming development [27]. A parallel example can be seen in the value of sexual and gender minority community involvement as a protective factor against various physical and mental health conditions [28,29]. The present study investigates several potential protective factors in this population, namely relationship orientation, demography, and information processing preferences. 

### 1.3. Relationship Orientation, Marginalized Identity, and Preferences in Information Processing Applied to Suicide

In the general population, married persons tend to fair better compared to divorced and single persons with respect to suicide risk [30,31]. However, this narrow definition of relationship status fails to capture the full scope of romantic involvement. Moreover, orientations to relationships (e.g., non-monogamy) remains understudied as a possible risk or protective factor for health in general. Of particular interest to the alternative sexuality community is non-monogamy, as opposed to other statuses (e.g., single/not dating, married, in a long-term committed relationship). Relationship orientations involving more than one partner are particularly common among members of the alternative sexuality community [32]. Although consensual non-monogamy has not been investigated with SRB to date, data exists to suggest potential rationales to think non-monogamy may function as either a risk or protective factor. Importantly, compared to persons in monogamous relationships, non-monogamous-identifying persons are more likely to identify as a sexual orientation minority, be divorced, and make less money [33]. Likewise, non-monogamous persons are more likely to endorse the range of sexually diverse identities [34]. Such multiple marginalized identities raise the possibility of an elevated risk for suicide compared to those of monogamous relationship orientations. However, Ferrer [35] summarized literature arguing that non-monogamy can also empower people to be autonomous and is predicated on authenticity, which may enhance health. Finally, a critical review of the literature concluded that persons with consensual non-monogamous orientations (i.e., polyamory, swinging, and others) report similar mental health compared to those in monogamous relationships [36]. Given the conflicting multiple minority versus empowerment and equitable health views, we explore whether non-monogamous relationship orientations are negatively or positively associated with SRB. 

Health equity frameworks [37] aim to redress health disparities through understanding demographic stratification and social determinants of health. This social justice view also holds that it is valuable to attend to the multitude of marginalized minority identities (e.g., race, sexual orientation, gender) to fully account for the variety of experiences within a community. Reflecting this perspective, multiple-minority identities may be a valuable perspective from which to examine risk and resilience in the alternative sexuality community. Indeed, a variety of intersecting identities play a role in the interest and engagement in alternative sexuality [38]. Such identities include a range of sexual orientation and gender diverse identities. A wealth of literature suggests the intersection of racial, sexual orientation, gender, and other multiple marginalized statuses is associated with a range of negative physical and mental health [28,39,40] and resilience [41,42,43] outcomes. Likewise, literature suggests that marginalized identities of sexual orientation minority (e.g., lesbian, gay, bisexual) and gender minority (e.g., transgender and gender non-conforming) are at higher risk for SRB compared to heterosexual and cisgender counterparts, respectively [44,45,46]. 

Increasingly, researchers are beginning to examine how holding multiple intersecting, marginalized identities may be useful in understanding SRB [47]. For instance, a recent systematic review of discrimination and mental health concluded that heterosexist prejudice, compared to racial prejudice, was most influential on SRB among sexual and gender minority (SGM) Black persons [48]. Also, another review of transgender and gender minority suicide showed that multiple marginalized statuses pertaining to race and religion were uniquely important factors among gender diverse persons [49]. Thus, we examined the SRB risk and protective factor patterns for the following set of marginalized identities among members of the alternative sexuality community: race, sexual orientation, gender, education level, and ethnicity. The present study is the first to do so among members of NCSF.

An emerging SRB model is the Preferences in information processing approach (PIP) [50]. According to PIP, individuals differ in the degree to which they are motivated to experience and process emotional and cognitive information in their environment. PIP can be considered a meta-theory of emotion and cognition grounded in the broader social–cognitive information processing theories [51,52]. PIP posits that individual differences in orientation to affect and cognition, respectively, may have direct and moderating influences on SRB [50]. Specifically, Need for Affect (NFA) [53,54], or one’s willingness to approach or avoid affective experiences and content, is thought to directly impact suicide as a risk factor to the extent to which one prefers to avoid emotionally-based experiences. Moreover, NFA is comprised of two facets, namely NFA Avoidance (i.e., extent to which one evades emotional experience and expression) and NFA Approach (i.e., degree to which one intentionally seeks affective experience and expression) [53,55]. Illustrative examples of each NFA construct are as follows. High affective avoidance may take the shape of positive views of or preference for refusing to discuss emotions (e.g., hopelessness, joy), engaging in emotionally numbing or blunting behaviors (e.g., substance use), or valuing facts and statistics over emotions in decision-making. On the other hand, higher orientation to engagement of affect may be observed in examples such as seeking out opportunity to discuss positive or negative feelings (e.g., in intimate relationships), expressing verbal or non-verbal gratitude or irritation with others, or processing emotions as a vital part of making decisions. 

Each aspect of NFA is hypothesized to function differentially as moderators of various suicide risk factors; for instance, for symptoms of depression or anxiety, higher NFA Avoidance may amplify suicide risk, whereas NFA Approach may mitigate such risk. Preferences in cognitive information processing are reflected in the individual difference of Need for Cognition (NFC) [56]. NFC encompasses one’s willingness to engage in flexible thinking and desire to approach or avoid specific thoughts and/or cognitive processes, rather than the presence of a particular cognitive process or rigid attributional style. Unlike NFA, however, NFC is typically measured uni-dimensionally [50]. The role of NFC is relatively understudied in the PIP-suicide model.

A series of studies [20,50,57,58] have applied the PIP to suicide risk in non-clinical samples. The most robust main effect observed to date concerns NFA Avoidance as a risk factor for suicide [50,55,58], possibly through association with the development of perceived burdensomeness and thwarted belongingness [57]. NFA and NFC have both shown sample-specific moderating roles. Elevated NFA Avoidance amplified the negative impact of depression and NFC on lifetime SRB among college students [50]. Among adults in the United Kingdom, NFA Approach mitigated the negative effects of depression on suicidal ideation, whereas NFC demonstrated mixed moderation patterns depending on the suicide risk factor [58]. Most germane to the current study, the only PIP model facet examined in a sample of NCSF members was NFA [20]. Application of these findings to NCSF members was tempered by the fact that findings were not disaggregated between the three samples in the study. This limitation notwithstanding, findings indicated that both NFA Approach and NFA Avoidance were risk factors for being categorized as having an elevated risk for suicide. These findings warrant replication and extension with all salient components of the model (i.e., inclusion of NFC). The present study fills this gap in the literature.

### 1.4. The Present Study

Emerging evidence suggests that SRB may be a unique concern for members of the alternative sexuality community. This study sought to fill the gap of identifying further risk and protective factors for SRB among NCSF members. We do so by identifying relationship orientation, multiple marginalized identities, and PIP factors differentiating individuals with non-suicidal, suicide ideation only, and suicide attempt histories. The following research questions (RQs) and hypotheses (Hs) were examined:

**H1**: *Multiple marginalized identities (i.e., racial, sexual orientation, gender, educational level, and ethnic minority NCSF members) will be associated with an elevated risk for more severe SRB. Alternatively, majority group-identifying NCSF members will be associated with a decreased risk for severe SRB.*

**H2**: *NFA Avoidance will be associated with an elevated risk for more severe SRB.*

**H3**: *NFA Approach will lessen the positive association between depression and SRB, thereby serving as a protective factor in the depression-suicide link.*

**RQ1**: *What is the association between relationship orientation (i.e., single/not dating, monogamous relationship, and polyamorous/open relationship) and SRB?*

**RQ2**: *How is NFC associated with SRB?*

## 2. Materials and Methods

### 2.1. Study Design and Procedure

Study inclusion criteria included: (1) 18 years of age or older, (2) NCSF membership, and (3) residing in the United States. No other specific inclusion/exclusion criteria were applied to the study. All participants provided informed consent for inclusion before they participated in the study. Participants gave electronic consent by clicking through to participate in the study after reading an informed consent page. Consent information summarized the purpose of the study, rights and limits of research participants, study team and Institutional Review Board (IRB) contact information, and incentive structure. Participants were debriefed at the end of the survey. The study was conducted in accordance with the Declaration of Helsinki, and the protocol was approved by the IRB/Ethics Committee of Old Dominion University (project ID code 1139007-4]). The present study used a single-time point online Qualtrics-administered survey. NCSF advertised the opportunity to complete an online survey regarding health and technology engagement via email to its listserv members (*n* = 6678) and social media accounts. Invalid email addresses were not tracked. Social media followers may be redundant with the listserv. A participation rate of 5.0% (334/6678) is the most accurate that can be tabulated, but may be an underestimate. Listserv members live across the United States. Participation in the survey was incentivized by offering participants the opportunity to enter a drawing for several e-gift cards. Data collection occurred over a five-month period between February and July 2018. 

### 2.2. Participants

Table 1 contains a summary of sample demographics. Comprised of 334 NCSF members, the sample was diverse with respect to sexual orientation, gender, and relationship status. However, the sample was primarily White and non-Hispanic/Latino(a), with more than half of participants possessing at least a bachelor’s degree. This participant breakdown is similar to the NCSF general membership [59] and prior NCSF survey studies [9]. Also, more than three-quarters of the sample reported a history of SRB. High prevalence rates of SRB are consistent with prior literature indicating that NCSF members are at an elevated clinical risk for suicide compared to general and college student adult samples [20].

### 2.3. Measures

#### 2.3.1. Demographics

Participants provided demographic information including race, gender, sexual orientation, relationship status, ethnicity, and education. Suicide exposure was assessed via two questions: knowing someone who died by suicide and knowing someone who attempted suicide. Both questions referenced lifetime suicide exposure. For each category, participants checked each of the following persons(s) they knew: an acquaintance, a friend, a family member, and other person. For this study, we created scores for suicide attempt exposure and suicide death exposure by summing the number of types of persons checked (possible range 0–4). 

#### 2.3.2. Suicide-Related Behavior

Participants completed the Suicidal Behaviors Questionnaire—Revised (SBQ-R) [18], a 4-item self-report questionnaire assessing lifetime and past-year suicidal behavior, communication of suicidal intent, and likelihood of future suicide attempt. Total scores were generated via summation, ranging from 3 to 18, with higher scores representing higher engagement in suicidal behavior. Item 1 of the SBQ-R generates lifetime SRB subgroups [18]; this approach to categorization of suicide-related behaviors has been used in prior studies [58,60,61]. Cut scores indicate individuals at clinical risk, with a score of seven or greater used among non-clinical adult samples [18]. In the current study, total scores and item one lifetime SRB subgroups were used. The SBQ-R demonstrated good internal consistency (α range = 0.87–0.97) [18]. In the current study, internal consistency was acceptable (α = 0.77).

#### 2.3.3. Preferences in Information Processing

Participants completed the Need for Cognition Scale short form [56], an 18-item self-report questionnaire assessing PIP beliefs related to thinking. Item responses were recorded on a 5-item scale ranging from 1 (“Extremely uncharacteristic of me”) to 5 (“Extremely characteristic of me”). Nine items were reverse coded before summing responses, with higher scores indicating greater engagement in and enjoyment of thinking. The NFC scale demonstrated good internal consistency [50,55], and among the current sample, the internal consistency of the NFC was also good (α = 0.85).

The Need for Affect Questionnaire Short Form (NAQ-S) [53] was used to assess PIP beliefs related to emotion. The NAQ-S is a 10-item self-report questionnaire with a 7-point response scale ranging from −3 (“Strongly disagree”) to 3 (“Strongly agree”). Subscale scores were generated via summation for NFA Approach (five items) and NFA Avoidance (five items), with higher scores indicating higher approach and avoidance of emotions, respectively. The NAQ-S demonstrated acceptable internal consistency for both NFA Approach (α range = 0.71 to 0.76) and NFA Avoidance (α range = 0.79 to 0.84) [53]. Internal consistency in the current study was good for both subcomponents (NFA Approach: α = 0.80; NFA Avoidance: α = 0.85).

#### 2.3.4. Mental Health

Participants completed the Depression Anxiety and Stress Scale short version (DASS-21) [62,63], a 21-item self-report questionnaire assessing past-week symptoms of depression (seven items), anxiety (seven items), and stress (seven items). Responses were scored on a 4-point Likert scale ranging from 0 (“Did not apply to me at all”) to 3 (“Applied to me very much, or most of the time”). Responses were summed such that higher sub-component scores indicated higher levels of depression, anxiety, and stress. In the current study, the depression and anxiety subscales were used. The DASS-21 demonstrates good internal consistency among non-clinical samples (depression: α = 0.88; anxiety: α = 0.82). Internal consistency in the current sample was similarly strong (depression: α = 0.92; anxiety α = 0.84).

### 2.4. Data Analysis

We used SPSS version 26.0 to assess hypotheses and research questions. Bivariate associations of demographics with SRB group status were examined via either chi-square or analysis of variance (ANOVA) analyses with effect size metrics of Cramer’s V and Cohen’s *d*, respectively. Effect size interpretation is guided by Cohen’s [64] effect size guidelines. These guidelines are: ±0.2 (small), ±0.5 (moderate), and ±0.8 (large). Multinomial logistic regression was employed to examine PIP and covariate associations with lifetime SRB group status. Continuous predictor variables were centered prior to analyses. Significant overall effects were subsequently examined for group differentiation of ideation only (reference group) from non-suicidal and suicide attempt comparison groups. Effect sizes are reported in odds ratios (ORs), with interpretation of magnitude of effects informed by guidelines in the statistical literature [65].

## 3. Results

Prior to hypothesis testing, several demographic factors required collapsing due to low cell counts (see Table 1). Race was recategorized as either White or racial minority. Sexual orientation was reclassified as either heterosexual or sexual minority. Gender was reconstituted as male, female, and transgender/gender non-conforming/gender non-binary (TGNC+). Educational subgroups of some high school and high school diploma/general education degree were combined due to the small cell size of the former.

### 3.1. Bivariate Analyses

Table 2 summarizes bivariate analyses of demographic, PIP, and mental health variables across lifetime SRB group. Answering RQ1, a large significant effect of relationship status was observed. The pattern was somewhat complex; being single was associated with highest frequency of suicide attempt group status, whereas non-monogamous and monogamous statuses were the most frequent in the ideation group. Monogamous relationship status was the highest frequency in the no SRB group. Consistent with H1, the following multiple marginalized (i.e., member of the alternative sexual community plus other marginalized status) patterns were observed. Frequencies for the following groups were elevated in the more severe lifetime SRB groups (i.e., ideation and attempt): (1) both TGNC+ and female compared to male, (2) sexual minority compared to heterosexual, and (3) lower education compared to higher education. The range of effect sizes for these effects were moderate–large. Contrary to H1, no significant racial and ethnic differences were observed. 

Supporting H2, NFA Avoidance was associated with lifetime SRB group status. Persons in the suicide attempt group reported significantly higher scores compared to no SRB (moderate effect) and ideation only group (small effect). No other PIP-related associations were observed. 

The following demographic and mental health related significant patterns were also observed: (1) lower suicide attempt exposure in the no SRB group compared to the ideation (small effect) and attempt (large effect) groups; (2) lower suicide death exposure in the ideation group compared to the suicide attempt group (small effect); and (3) identical patterns of increasing total suicide-related behavior, depression, and anxiety across no SRB, ideation, and suicide attempt groups (moderate–large effects). In light of bivariate analyses, participant gender, sexual orientation, education, relationship orientation, suicide attempt exposure, and suicide death exposure were retained as covariates for full PIP model testing. Answering RQ2, no bivariate association was observed between NFC and SRB groups.

### 3.2. Multi-Nomial Regression Model Testing Covariate and PIP Effects on Lifetime SRB Group Status

The multi-nomial model featured the following analytic parameters: (1) lifetime SRB group status was the dependent measure (ideation as reference group in order to identify factors differentiating three-step suicide theory groups); (2) covariates of gender (TGNC+ as reference group), sexual orientation (sexual minority as reference group), education level (graduate degree as reference group), relationship status (single as reference group), suicide attempt exposure, and suicide death exposure; (3) main effects for depression, anxiety, NFA Avoidance, NFA Approach, and NFC, and; (4) two-way PIP-supported interactions of anxiety x NFA Avoidance, anxiety x NFA Approach, anxiety x NFC, depression x NFA Avoidance, depression x NFA Approach, depression x NFC, NFA Avoidance x NFA Approach, NFA Avoidance x NFC, and NFA Approach x NFC.

Table 3 contains summary statistics for the overall effects of each predictor multi-nomial regression model. Where significant effects emerged with categorical predictors, follow-up inspection of group comparisons are also reported. The model displayed good fit to the data, χ^2^ (618) = 623.05, *p* = 0.44. Overall, the collection of predictors accounted for significant and large variance in lifetime SRB group status, χ2 (48) = 153.87, *p* < 0.001, Cox and Snell *R*^2^ = 0.37, Nagelkerke *R*^2^ = 0.43. Contrary to H1, significant effects were not observed for gender or sexual orientation. Contrary to H2, a significant effect was not observed for NFA Avoidance. Significant overall effects requiring follow-up inspection were observed for (1) relationship orientation, (2) education level, (3) anxiety, (4) depression, and (5) depression x NFA Approach. Regarding RQ1, compared to being in a non-monogamous relationship, being in a monogamous relationship was associated with increased odds (B = 0.68, seB = 0.34, Wald χ2 (1) = 3.97, *p* = 0.046, OR = 1.97, 95% confidence interval (CI) = 1.01–3.83) of no SRB group status. Further, compared to being in a non-monogamous relationship, being in a monogamous relationship was associated with decreased odds (B = −1.11, seB = 0.44, Wald χ2 (1) = 6.34, *p* = 0.01, OR = 0.33, 95% CI = 0.14–0.78) of suicide attempt group status. 

Regarding H2, compared to persons with a graduate degree, both persons with some high school/general education degree (B = 1.12, seB = 0.51, Wald χ2 (1) = 4.79, *p* = 0.03, OR = 3.05, 95% CI = 1.12–8.29) or an associate’s degree (B = 1.03, seB = 0.51, Wald χ2 (1) = 4.00, *p* = 0.04, OR = 2.80, 95% CI = 1.02–7.68) were associated with increased odds of suicide attempt group status. Concerning mental health main effects, increases in anxiety were associated with increased odds of suicide attempt group status (B = 0.68, seB = 0.25, Wald χ2 (1) = 7.42, *p* = 0.006, OR = 1.97, 95% CI = 1.21–3.22). Decreases in depression were associated with increased odds of no SRB group status (B = −1.24, seB = 0.35, Wald χ2 (1) = 12.29, *p* < 0.001, OR = 0.29, 95% CI = 0.14–0.58). 

**Figure 1:**
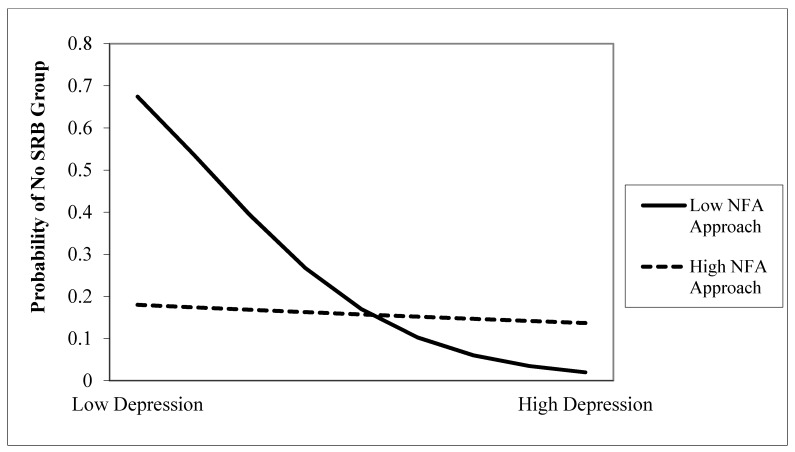
Two-way interaction of depression x Need for Affect Approach predicting no suicide-related behavior group status (ideation reference group). Notes: SRB = Suicide-related behavior; NFA = Need for Affect; Low/High = ±1 standard deviation around the mean.

Assessing H3, this depression main effect was qualified by a depression x NFA Approach interaction (B = 1.08, seB = 0.45, Wald χ2 (1) = 5.75, *p* = 0.02, OR = 2.93, 95% CI = 1.22–7.08). The pattern of the interaction suggests that, compared to ideation group status, the odds of no SRB group status drops as depression increases, but only for those high in NFA Approach (see Figure 1). In other words, NFA Approach buffers the likelihood of ideation group status in the context of depression.

#### Multinomial Model Summary

Monogamous, as opposed to a non-monogamous, relationship orientation was a small protective factor for SRB (RQ1). Possessing a graduate degree was a moderately-sized protective factor for suicide attempt (H1). NFA Avoidance was unrelated to SRB group status (H2). Increased anxiety was a small risk factor for suicide attempt (covariate finding). Increased depression was a moderately-sized risk factor for suicidal ideation (covariate finding). NFA Approach was moderately-sized protective factor in the depression–suicidal ideation association (H3). Other H1 patterns were unsupported. NFC displayed no meaningful association with SRB in the present study (RQ2).

## 4. Discussion

SRB is a documented mental health problem in the alternative sexuality community [20], with existing literature highlighting mental health [21], stigma-associated emotions [13], and acquired capability [12] as risk factors. The present study employed relationship orientation (e.g., non-monogamy) [34,66], health inequity/marginalized identity [5,37], and PIP [50,58] to further investigate factors associated with SRB among members of the alternative sexuality community. As is discussed in the following sections, all three perspectives, plus known mental health and suicide exposure-related factors, added value in understanding SRB. This is an important conceptual statement on the nature of SRB among this vulnerable, yet diverse, population. It appears that no one tested theoretical model can fully explain the heightened SRB risk among members of NCSF. 

The present study was the first to our knowledge to explore non-monogamy and SRB. Appropriately framed as an exploratory research question (see RQ1), we observed an intricate pattern of one’s stated relationship orientation and SRB. Overall, it appears that being in a monogamous relationship is a protective factor. Yet, non-monogamous orientation was associated with almost half the suicide attempt frequency compared to being single. There are a few potential explanations for these patterns. First, given social support is a robust SRB protective factor [67,68], the support inherent in many monogamous relationships may explicate its protective role. A second explanation is that non-monogamous orientations, compared to monogamous ones, tend to covary with self-identification as a member of other marginalized communities [33,34]. The multitude of stigmatized identities may explain why non-monogamy, compared to monogamy, is an SRB risk factor. The possibility also exists that the answer to this pattern is more complex. Societal norms favor marriage or other monogamous couples compared to non-monogamy. Similar to the sexual and gender minority concept of identity concealment [69], it may be that persons with non-monogamous orientations must conceal this aspect of their identity from co-workers, family, and friends. Such concealment may be motivated by a number of reasons, such as fear of stigma and discrimination, or internalization of monogamous-normative belief systems. The potential of identity concealment among non-monogamous persons to facilitate suicide risk represents an avenue worthy of future research. 

Bivariate patterns were supported with respect to sexual and gender minority identities and SRB, mirroring broader sexual and gender minority and general population health literatures [28,39,48]. The impact of marginalized identities on risk, however, dropped out when considered simultaneously with other factors, giving way to the influence of lower education among others. Lower education, also not a biologically-based or inherent minority status, is connected to numerous health disparities [70,71], suggesting it is a factor fitting well into contemporary notions of marginalization. Importantly, research on the alternative sexuality community overall [12,34], and NCSF in particular [9], is restricted with respect to education status, with lower education levels being underrepresented. Other aspects of demography not accounted for in the present study (e.g., socioeconomic status) may explain education’s prominent role in SRB. On the other hand, increased resources, social capital, and other benefits resulting from attainment of higher education may also account for the protective role of higher degrees in the present study.

NFA-related findings largely replicated prior PIP studies of suicide. For instance, bivariate NFA Avoidance patterns are consistent with prior findings [50,55,58]. Interestingly, when the entire PIP model is accounted for (i.e., NFA and NFC main and moderating effects), NFA Approach was the only PIP-supported hypothesis. Specifically, NFA Approach served as a protective factor in limiting the depression-associated odds associated with suicidal ideation. A similar pattern was observed among community-dwelling adults in the United Kingdom in which NFA Approach buffered depression-associated odds of suicide attempt status [58]. High NFA Approach consists of an orientation toward willingness to engage with emotions, both positive and negative [54]. Such a willingness may facilitate better handling of depression, thereby mitigating SRB risk. Importantly, these findings extend PIP suicide theory by: (a) highlighting NFA Approach as a cross-culturally relevant suicide risk factor, (b) accounting for NFC in the models, and (c) examining PIP-informed hypotheses and questions in a solely alternative sexuality sample for the first time.

Consistent with prior literature [27,55,67,72], suicide exposure, anxiety, and depression were associated with more severe SRB. Notably, one prior study of sexual orientation minority identifying NCSF members reported similarly robust patterns of well-documented suicide risk factors (e.g., depression, post-traumatic stress [21]. While the present study advanced investigation of SRB factors that may be population-specific (e.g., polyamory), mental health findings from our study reinforce the notion that it remains important to attend to the most robust suicide-related factors as well. 

### Implications for Research, Health Education, and Suicide Prevention

Further research is needed to fully grasp the nature of SRB among members of the alternative sexuality community. The next steps in this research agenda should address a few necessary questions. First, relationship orientation should be investigated in depth in order to comprehend the nuance involved in SRB. Qualitative interviews or methods involving multi-partner comparison groups may provide such insight. Second, most PIP and alternative sexuality mental health research to date is cross-sectional in design. A prospective assessment of competing suicide-related theories such as the Interpersonal Theory of Suicide [19] and PIP [50,58] would be an invaluable next step. Such longitudinal work should account for both the demographic diversity (e.g., sexual and gender diversity) and restrictions (e.g., race, educational level) in its sampling and design. Stigma-based experiences also represent a relatively untapped pathway to SRB that is worthy of inquiry. 

Public and mental health implications of our findings pertain most directly to health education and healthcare provision for members of the alternative sexuality community. For example, a portion of NCSF’s stated mission is to promote health and equity for its membership [1]. One strategy to do so may be seen in the form of mental health education materials. Such psycho-educational materials may be web-based, provide interactive opportunities, and address key factors associated with SRB (e.g., avoidance or approach of affect, depression, education level). Existing evidence demonstrates that members of the alternative sexuality community frequently experience misunderstanding and discrimination from healthcare providers [59,73], yielding recent calls for BDSM/kink/polyamory-aware health service providers [74,75]. That is, a lack of understanding on the part of healthcare providers regarding BDSM/kink/polyamory definitions, culture, and health can convey stigma and result in negative experiences for members of the alternative sexuality community. Design of such training should include SRB as a key health indicator. Further, the unique demographic make-up, overall health nature of the population, mental health conditions associated with SRB, and roles of NFA Avoidance and NFA Approach could be appropriately highlighted. In instances of suicide-specific psychotherapy for persons identifying as members of this community, mental health professionals should account for the unique and general factors associated with SRB. For example, within the context of Collaborative Assessment and Management of Suicide (CAMS) [76], therapists can incorporate assessment and appropriate treatment that targets documented risk and protective factors such as relationship orientation, NFA, shame, guilt, depression, and anxiety.

## 5. Conclusions

This study contains several limitations necessary to acknowledge. With regard to sampling, electronic convenience sampling and a low participation rate limit the scope of capturing the full BDSM, kink, and polyamory communities. Likewise, the NCSF sample is restricted with regard to race and ethnicity. Generalizability is therefore restricted; future research should use varied sampling strategies to obtain more robust, representative, and diverse samples. Likewise, comparisons to general population samples would help identify truly unique risk and protective factors among members of the alternative sexuality community. Measurement limitations such as self-report data and discrete classification of lifetime SRB also limit confidence in the strength of findings. Ideally, other formats of SRB measurement, like clinician ratings or health records, can be integrated into future studies regarding the alternative sexuality community and suicide. Alternatively, as recent evidence suggests suicide deaths may not vary by key factors in our study (e.g., sexual orientation) [77], it appears critical to extend the present line of inquiry to suicide deaths. Finally, cross-sectional design is a methodological limitation of the entire SRB literature pertaining to the alternative sexuality community. Especially where theory testing and identification of mediation pathways are of interest, future research in this area would benefit from prospective methods capturing data via daily diary, electronic momentary assessment, and other innovative methodologies. 

SRB remains a pressing health concern for members of the alternative sexuality community. The present study advanced understanding of factors associated with SRB. Non-monogamous and monogamous relationship orientations appear to be important considerations in SRB for this group. However, further research is necessary to illuminate whether observed relationship orientation patterns are merely a function of other unaccounted for demography, or whether the nature of relationships in an alternative sexuality population inculcates unique risk and protective factor pathways to SRB. At most, we can conclude at this stage that monogamous status appears to be important for any population with respect to suicide, whereas non-monogamous orientations warrant further nuanced examination. Marginalized identity patterns were observed with respect to SRB risk, with education level mattering under conditions of simultaneous examination of various SRB factors. Lower education appears to be a general suicide risk factor at present. What is most important for the alternative sexuality community is for future health education and suicide prevention efforts to enhance educational and associated opportunities for those with limited educational and economic means. Evidence builds for NFA Avoidance as a ubiquitous SRB risk factor, whereas NFA Approach may be a depression-specific protective factor. Health education materials, BDSM/kink-aware healthcare provider training, and suicide-specific psychotherapeutic interventions may do well to account for the role of NFA moving forward. Finally, although expected, depression, anxiety, and suicide exposure were also associated with SRB. Again, these are consistent with broader suicide literature. It is important, however, not to lose sight of addressing the most robust SRB risk factors in prevention and intervention programming as well. 

## Figures and Tables

**Table 1:** Sample Demographics.

Variable	*n* (%)
Lifetime suicide-related behavior	
None	75 (22.5%)
Ideation only	190 (56.9%)
Attempt	69 (20.7%)
Race	
White	285 (85.3%)
Black	5 (1.5%)
American Indian/Alaskan Native	1 (0.3%)
Asian American	2 (0.6%)
Native Hawaiian/Pacific Islander	2 (0.6%)
Biracial	30 (9.0%
Other	9 (2.7%)
Sexual Orientation	
Gay	16 (4.8%)
Lesbian	4 (1.2%)
Queer	17 (5.1%)
Straight	73 (21.9%)
Questioning	1 (0.3%)
Experimenting	8 (2.4%)
Pansexual	45 (13.5%)
Demisexual	2 (0.6%)
Heteroflexible	27 (8.1%)
Bisexual	63 (18.9%)
Other	2 (0.6%)
Multiple sexual identities	76 (22.8%)
Education	
Some high school	4 (1.2%)
High school diploma/general education degree	61 (18.3%)
Associate’s Degree	56 (16.8%)
Bachelor’s Degree	99 (29.6%)
Graduate Degree	114 (34.1%)
Gender	
Male	117 (35.0%)
Female	172 (51.5%)
Male-to-female	7 (2.1%)
Female-to-male	4 (1.2%)
Transitioning	1 (0.3%
Queer	20 (6.0%)
Other	13 (3.9%)
Ethnicity	
Non-Hispanic/Latino(a)	320 (95.8%)
Hispanic/Latino(a)	14 (4.2%)
Relationship Status	
Single	35 (10.5%)
In a monogamous/long-term committed/married/civil union	113 (33.8%)
Non-monogamous	186 (55.7%)
Lifetime Suicide Death Exposure	
No	97 (29.0%)
Yes	237 (71.0%)
Lifetime Suicide Attempt Exposure	
No	59 (17.7%)
Yes	275 (82.3%)

Notes: *N* = 334.

**Table 2:** Lifetime suicide-related behavior group variation in demographics, preferences in information processing, and mental health.

Variable	Total Sample (*N* = 334)	None (*n* = 75)	Ideation Only (*n* = 190)	Attempt (*n* = 69)	Test-Statistic (df)	Effect Size
Gender					Χ2 (4) = 12.36, *p* = 0.01	0.19
Male	117	38 (32.5%)	61 (52.1%)	18 (15.4%)		
Female	172	32 (18.6%)	100 (58.1%)	40 (23.3%)		
TGNC+	45	5 (11.1%)	29 (64.4%)	11 (24.4%)		
Education					Χ2 (6) = 18.70, *p* = 0.005	0.24
Some high school/GED	65	11 (16.9%)	35 (53.8%)	19 (29.2%)		
Associate’s degree	56	9 (16.1%)	32 (57.1%)	15 (26.8%)		
Bachelor’s degree	99	17 (17.2%)	59 (59.6%)	23 (23.2%)		
Graduate degree	114	38 (33.3%)	64 (64.9%)	12 (10.5%)		
Sexual Orientation					Χ2 (2) = 7.53, *p* = 0.02	0.15
Straight/heterosexual	73	25 (34.2%)	36 (49.3%)	12 (16.4%)		
Sexual minority	261	50 (19.2%)	154 (59.0%)	57 (21.8%)		
Relationship Orientation					Χ2 (4) = 19.45, *p* = 0.01	0.24
Single	35	6 (17.1%)	15 (42.9%)	14 (40.0%)		
Monogamous Rel.	113	36 (31.9%)	64 (56.6%)	13 (11.5%)		
Non-monogamous Rel.	186	33 (17.7%)	111 (59.7%)	42 (22.6%)		
Race					Χ2 (2) = 0.84, *p* = 0.66	0.05
White	285	66 (23.2%)	162 (56.8%)	57 (20.0%)		
Racial minority	49	9 (18.4%)	28 (57.1%)	12 (24.5%)		
Ethnicity					Χ2 (4) = 2.12 *p* = 0.35	0.08
Non-Hispanic/Latino(a)	320	72 (22.5%)	184 (57.5%)	64 (20.0%)		
Hispanic/Latino(a)	14	4 (21.4%)	6 (42.9%)	5 (35.7%)		
Suicide attempt exposure	1.29 (0.96)	0.99 (0.89) a, b	1.27 (0.90) a	1.68 (1.06) b	F (2, 331) = 8.75, *p* < 0.001	a.31, b.70
Suicide death exposure	0.92 (0.78)	0.87 (0.83)	0.86 (0.72) a	1.13 (0.87) a	F (2, 331) = 3.28, *p* = 0.04	a.34
Total SRB	7.13 (3.47)	3.51 (0.93) a, b	7.17 (2.62) a,c	10.97 (3.05) b,c	F (2, 331) = 165.85, *p* < 0.001	a1.86, b3.31, c1.34
Depression	5.25 (5.15)	2.19 (2.50) a,b	5.51 (4.89) a, c	7.87 (6.30) b,c	F (2, 331) = 25.78, *p* < 0.001	a.85, b1.18, c.42
Anxiety	4.05 (4.15)	2.05 (2.49)	4.00 (3.86)	6.35 (5.15)	F (2, 331) = 21.60, *p* < 0.001	a.60, b1.06, c.52
NFA Approach	7.28 (5.41)	6.68 (5.75)	7.71 (5.05)	7.28 (5.41)	F (2, 331) = 1.37, *p* = 0.25	-
NFA Avoidance	−4.70 (7.62)	−7.07 (6.33) a	−4.71 (7.34) b	−2.07 (8.81) a,b	F (2, 331) = 8.04, *p* < 0.001	a.65, b.32
Need for Cognition	70.45 (10.20)	71.93 (11.28)	69.94 (9.66)	70.23 (10.40)	F (2, 331) = 1.04, *p* = 0.35	-

Notes: Chi-square tests column statistics = count (%); F-test column statistics = Mean (standard deviation); Suicide-related behavior (SRB) group = Lifetime none, ideation only, or attempt based on SBQ-R item 1 [18]; df = Degrees of freedom; Effect Size = Cramer’s V for chi-square analysis, Cohen’s *d* for significant differences in SRB group categories; GED = general education degree; Identical subscripts in same column = significant difference in Bonferroni post-hoc test; TGNC+ = Transgender, Gender Non-Conforming, and Gender Non-Binary; Rel. = Relationship; NFA = Need for Affect.

**Table 3:** Multi-nomial regression model of lifetime suicide-related behavior group status.

Predictor	F (df)	*p*-value
Intercept	-	-
Gender	1.72 (4)	0.79
**Education level**	**12.95 (6)**	**0.04**
**Relationship orientation**	**18.52 (4)**	**0.001**
Sexual orientation	2.68 (2)	0.26
Suicide attempt exposure	5.21 (2)	0.07
Suicide death exposure	5.21 (2)	0.07
**Anxiety**	**8.46 (2)**	**0.01**
**Depression**	**15.71 (2)**	**<0.001**
Need for Affect Approach	0.03(2)	0.98
Need for Affect Avoidance	0.20(2)	0.90
Need for Cognition	0.69(2)	0.71
Anxiety x Need for Affect Approach	4.54 (2)	0.10
Anxiety x Need for Affect Avoidance	0.91 (2)	0.63
Anxiety x Need for Cognition	4.23 (2)	0.12
**Depression x Need for Affect Approach**	**10.93 (2)**	**0.004**
Depression x Need for Affect Avoidance	1.06 (2)	0.59
Depression x Need for Cognition	5.31 (2)	0.07
Need for Affect Approach x Need for Affect Avoidance	1.16 (2)	0.56
Need for Affect Approach x Need for Cognition	1.45 (2)	0.48
Need for Affect Avoidance x Need for Cognition	1.30 (2)	0.52

Notes: x = Interaction term; Lifetime suicide-related behavior group status = None, ideation only (reference group), and attempt; **Bold** font denotes significant predictor; *N* = 334.
